# LRP1 expression in colon cancer predicts clinical outcome

**DOI:** 10.18632/oncotarget.24225

**Published:** 2018-01-13

**Authors:** Camille Boulagnon-Rombi, Christophe Schneider, Chloé Leandri, Albin Jeanne, Virginie Grybek, Aude Marchal Bressenot, Coralie Barbe, Benjamin Marquet, Saviz Nasri, Christelle Coquelet, Caroline Fichel, Nicole Bouland, Arnaud Bonnomet, Reza Kianmanesh, Anne-Sophie Lebre, Olivier Bouché, Marie-Danièle Diebold, Georges Bellon, Stéphane Dedieu

**Affiliations:** ^1^ Laboratoire de Biopathologie, Centre Hospitalier Universitaire et Faculté de Médecine, Reims, France; ^2^ CNRS UMR 7369, Matrice Extracellulaire et Dynamique Cellulaire, MEDyC, Reims, France; ^3^ Université de Reims Champagne-Ardenne, UFR Sciences Exactes et Naturelles, Campus Moulin de la Housse, Reims, France; ^4^ Service de Gastro-entérologie et Cancérologie Digestive, Centre Hospitalier Universitaire, Reims, France; ^5^ SATT Nord, Lille, France; ^6^ Laboratoire de Génétique, Centre Hospitalier Universitaire, Reims, France; ^7^ Unité d’Aide Méthodologique, Centre Hospitalier Universitaire, Reims, France; ^8^ CRB Tumorothèque de Champagne-Ardenne, Reims, France; ^9^ Plateforme d’Imagerie Cellulaire et Tissulaire, Université de Reims Champagne-Ardenne, Reims, France; ^10^ Service de Chirurgie Digestive, Centre Hospitalier Universitaire, Reims, France; ^11^ Laboratoire de Biochimie, Centre Hospitalier Universitaire, Reims, France

**Keywords:** colorectal cancer, LRP1, miR-205, BRAF, microsatellite instability, Pathology

## Abstract

LRP1 (low-density lipoprotein receptor-related protein 1), a multifunctional endocytic receptor, has recently been identified as a hub within a biomarker network for multi-cancer clinical outcome prediction. As its role in colon cancer has not yet been characterized, we here investigate the relationship between LRP1 and outcome.

**Materials and Methods:**

*LRP1* mRNA expression was determined in colon adenocarcinoma and paired colon mucosa samples, as well as in stromal and tumor cells obtained after laser capture microdissection. Clinical potential was further investigated by immunohistochemistry in a population-based colon cancer series (*n* = 307). *LRP1* methylation, mutation and miR-205 expression were evaluated and compared with LRP1 expression levels.

**Results:**

*LRP1* mRNA levels were significantly lower in colon adenocarcinoma cells compared with colon mucosa and stromal cells obtained after laser capture microdissection. Low LRP1 immunohistochemical expression in adenocarcinomas was associated with higher age, right-sided tumor, loss of CDX2 expression, Annexin A10 expression, CIMP-H, MSI-H and *BRAF*V600E mutation. Low LRP1 expression correlated with poor clinical outcome, especially in stage IV patients. While LRP1 expression was downregulated by *LRP1* mutation, *LRP1* promoter was never methylated.

**Conclusions:**

Loss of LRP1 expression is associated with worse colon cancer outcomes. Mechanistically, *LRP1* mutation modulates LRP1 expression.

## INTRODUCTION

Colorectal cancer (CRC) is the third most common cancer diagnosed worldwide in men and the second in women. Despite advances in screening, diagnosis and management of the disease, it remains the fourth cancer in terms of mortality. Metastatic disease ultimately occurs in approximately 50–70% of patients presenting colorectal cancer [1–3]. UICC staging is the only prognostic classification used in clinical practice to select patients for adjuvant chemotherapy [4]. Currently, CRC has relatively few established biomarkers to predict patient outcome. Molecular markers include microsatellite instability (MSI), *RAS* and *BRAF* mutation. *RAS* and *BRAF* mutation status are used to guide therapeutic decisions in metastatic CRC patients. CRC with *RAS* or *BRAF* mutations are unlikely to respond to anti-epidermal growth factor receptor (EGFR) antibody therapy [5–7]. Patients with nonhereditary MSI tumors have better prognosis than those with microsatellite stable (MSS) tumors [1, 2, 8–10], and MSI is currently implemented in clinical guidelines as a prognostic biomarker, especially in stage II CRC patients [11]. However, these histomolecular parameters hardly apprehend disease heterogeneity and are insufficient for recurrence and prognostic prediction in an individual patient. Therefore, robust biomarkers that can stratify patient prognosis groups and improve treatment strategies are urgently needed.

The low-density lipoprotein receptor (LDLR)-related protein-1 (LRP1), a member of LDLR family, is a large multifunctional endocytic cell surface receptor, which is ubiquitously expressed [12, 13]. This large transmembrane receptor recognizes numerous ligands, therefore regulating a wide range of biological functions. It both acts as a signaling and clearance receptor. The biological activity of LRP1 was initially characterized as a clearance receptor for chylomicron remnants and complexes of α_2_-macroglobulin with proteinases [14]. Subsequent work has revealed that this receptor regulates the cell ability to respond to growth factors, to interact with extracellular matrix, as well as to respond to perturbations that occur within the microenvironment [15–18]. Numerous studies have suggested a role for LRP1 in regulation of tumor growth and progression. LRP1 has recently been identified as a hub within a biomarker network for multi-cancer clinical outcome prediction [19]. However, the role of LRP1 varies from one tumor type to another. Indeed, several studies have reported that low LRP1 expression was closely related to advanced tumor stages and poor survival in several solid tumors, such as hepatocellular carcinoma [20], lung adenocarcinoma [21] melanoma [22] and Wilms tumors [23]. On the contrary, high LRP1 expression was related to advanced tumor stages in endometrial carcinoma [24], breast cancer [25] and prostate carcinomas [26]. Using *in vitro* models, it has been demonstrated that LRP1 neutralization could abrogate cell motility in both tumor and non-tumor cells, and this despite an increase in pericellular proteolytic activities of several extracellular proteases such as MMP2 (Matrix Metalloproteinase 2), MMP9 and uPA (urokinase Plasminogen Activator) [20, 27]. On the other hand, LRP1 silencing prevents spread of glioblastoma cells [28]. Therefore, LRP1 influence on tumor cell migration and invasion likely depends on the tumor cell type and the specific extracellular proteins involved in these processes [29].

In CRC, little is known about LRP1 and its putative function. Previous studies on few colon adenocarcinomas samples showed a frequent loss of LRP1 immunohistochemical expression in adenocarcinomatous cells [30, 31]. To further expand our knowledge on the relevance of considering LRP1 expression in colon cancer, we analyzed LRP1 expression level and distribution in a series of 307 colon cancers with follow-up data. We then determined whether LRP1 expression is linked to clinical characteristics and outcomes while analyzing the role of miRNA expression, *LRP1* mutation and methylation in LRP1 expression profile.

## RESULTS

### Patients and clinicopathological features

In total, 307 colon cancer patients were included in our study. The population comprised 174 (57%) men and 133 (43%) women, whose mean age was 71 years (± 11 years). Tumors were right-sided in 136 cases (44%) and left-sided in 171 cases (56%). Follow-up data were available for all except 12 patients. The mean follow-up time was 43 months (± 32 months). Clinicopathological features of the cohort are detailed in Table 1.

**Table 1:** Clinicopathological features of the cohort

Clinical/pathological features	No. available (%)
**Gender**	
Male	174 (57)
Female	133 (43)
**Mean age [range]**	71 years [41-91 years]
**UICC stage**	
Stage I	35 (11)
Stage II	117 (38)
Stage III	79 (26)
Stage IV	76 (25)
**Tumor location**	
Left colon	171 (56)
Right colon	136 (44)
**Occlusion**	
Yes	35 (11)
No	272 (89)
**Tumor perforation**	
Yes	20 (7)
No	287 (93)
**Differentiation grade**	
Grade 1–2	258 (84)
Grade 3	49 (16)
***KRAS* status**	
Wild type	101 (68)
Mutant	48 (32)
***BRAF* status**	
Wild type	260 (86)
Mutant	44 (14)
**Microsatellite status**	
MSS	266 (87)
MSI	40 (13)
**CIMP status**	
No CIMP	22 (34)
CIMP-Low	35 (55)
CIMP-High	7 (11)

### LRP1 is lower expressed in adenocarcinoma cells compared with normal colon mucosa and stromal cells

**Figure 1:**
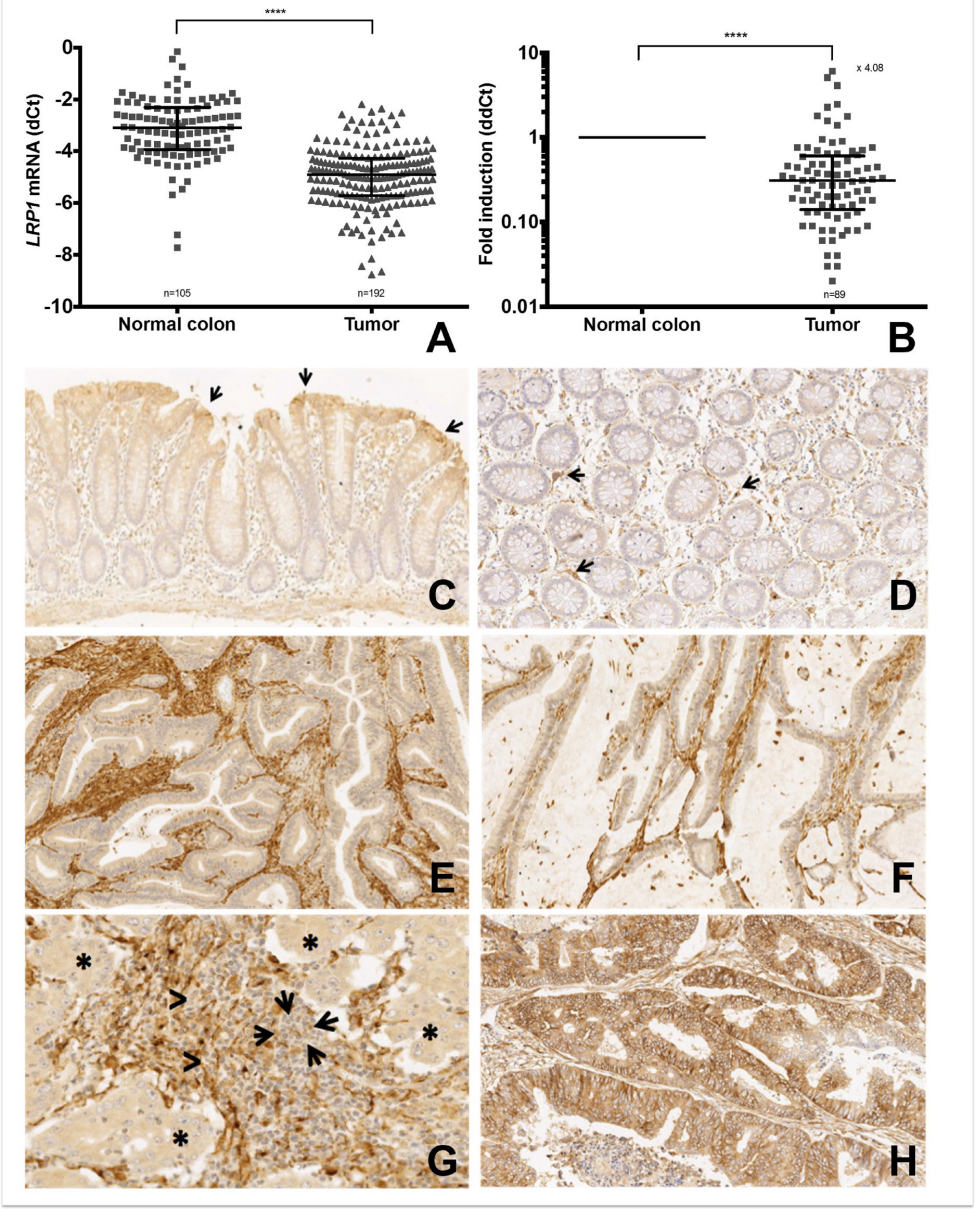
LRP1 expression in colon cancer cells compared to normal colon and stromal cells (**A**) qRT-PCR expression levels of *LRP1* mRNA in colon adenocarcinoma fresh frozen samples compared with normal colon mucosa fresh frozen samples. Values are shown as dCt normalized with *RPL32* (**B**) Comparative quantification analysis of *LRP1* mRNA expression levels in tumor samples compared with paired normal colon mucosa samples. Values are shown as ddCt fold induction. ^****^p < 0.0001, Mann Whitney test. (**C–H**) Representative microphotographs of LRP1 immunohistochemistry on colon mucosa (C–D) and colon adenocarcinoma (E–H). (C) LRP1 expression in surface epithelium (arrows) in normal colon mucosa (×5 magnification). (D) LRP1 expression in fibroblasts of the lamina propria (arrows) in normal colon mucosa (×10 magnification). Loss of LRP1 expression in malignant cells of a moderately differentiated adenocarcinoma (E) and a mucinous adenocarcinoma (F) (×20 magnification). (G) Loss of LRP1 expression in malignant cells (asterisks) and stromal lymphocytes (arrows) of a poorly differentiated adenocarcinoma (×30 magnification). (H) LRP1 expression in malignant and stromal cells of a moderately differentiated adenocarcinoma (×20 magnification).

*LRP1* mRNA expression analyses by quantitative real-time reverse transcriptase polymerase chain reaction (qRT-PCR) on 192 colonic adenocarcinoma samples and 105 colonic mucosa samples with RQI values ≥5 showed a 4.08-fold decrease in LRP1 expression within tumor samples when compared with normal colon samples (Figure 1A and 1B). LRP1 was overexpressed when compared with paired normal colon samples in only 9/85 adenocarcinoma cases (10.6%; data not shown).

To describe LRP1 distribution in colon tissues, immunohistochemical (IHC) analyses were performed on paired normal colon mucosa samples (*n* = 117) and colon adenocarcinomas (*n* = 307). In colon mucosa, epithelial cells expressed LRP1 in 86% cases (101/117). In the majority (85/101) of these cases, LRP1 expression was limited to surface epithelium (Figure 1C). Some fibroblasts of the lamina propria expressed LRP1 (Figure 1D). In adenocarcinoma, LRP1 was expressed in malignant cells in 244/307 (79%) of the cases. The mean IHC tumor score was 6.22 ± 3.62. In these adenocarcinoma samples, stromal fibroblasts expressed LRP1 in all cases (Figure 1E–1H). The mean IHC stroma score was near optimal (10.82 ± 2.44). LRP1 was never found to be expressed in stromal lymphocytes (Figure 1G). Immunohistochemical expression of LRP1 was inversely correlated in malignant and stromal cells (*p* = 0.0003; *R*^2^ = 0.04). We didn’t find any difference of IHC scores between the center and the invasive front of the adenocarcinomas for both tumor and stromal cells.

Furthermore, IHC analyses performed on 14 conventional adenomas (8 low-grade, 6 high-grade) revealed that LRP1 IHC score was significantly higher in adenoma cells when compared with adenocarcinoma cells (data not shown), and this whatever the grade.

### Microdissection analyses confirm low *LRP1* mRNA expression in malignant cells compared to stromal cells

**Figure 2:**
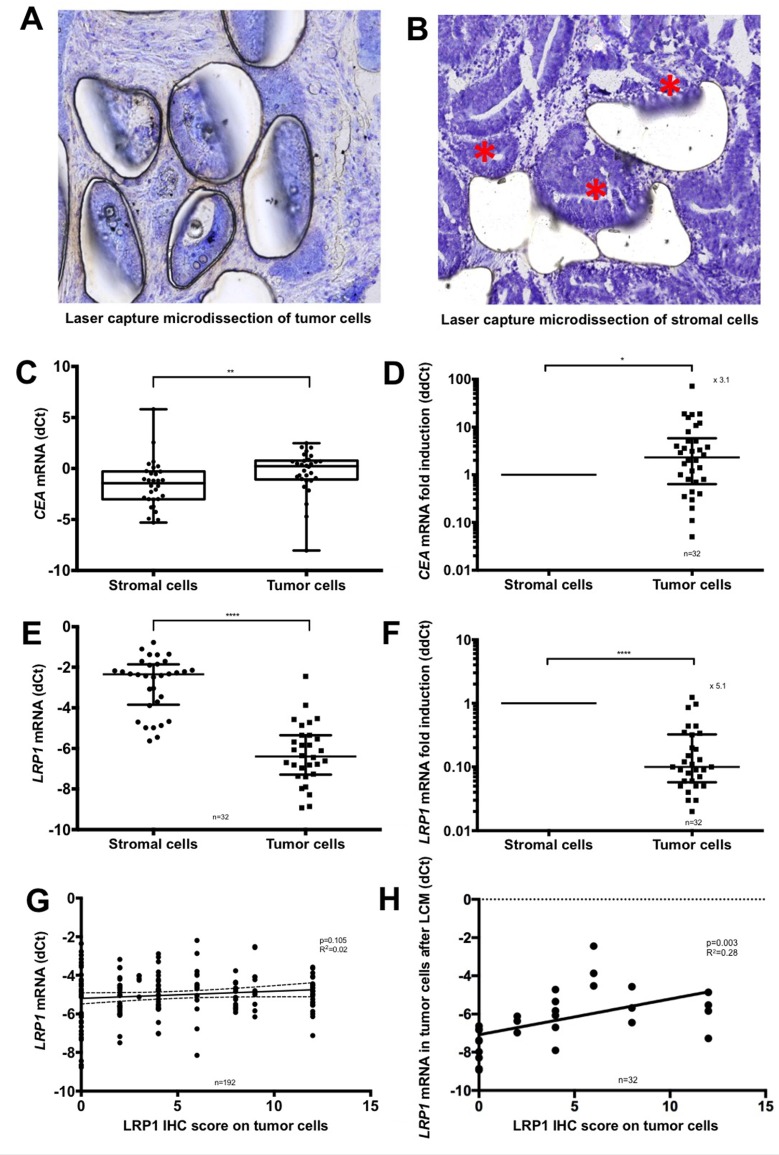
Laser capture microdissection analyses (**A–B**) Representative microphotographs of microscopic control of laser capture microdissection (LCM) (Cresyl violet, ×20 magnification). (A) Microdissection of the malignant cells. (B) Microdissection of the stromal cells. Residual malignant glands are highlighted with an asterisk. (**C**) qRT-PCR expression levels of *CEA* mRNA in adenocarcinoma cells compared with stromal cells after LCM. Values are shown as dCt normalized with *RPL32*. (**D**) Comparative quantification analysis of *CEA* mRNA expression levels in tumor cells compared with stromal cells after LCM. Values are shown as ddCt fold induction. (**E**) qRT-PCR expression levels of *LRP1* mRNA in adenocarcinoma cells compared with stromal cells after LCM. Values are shown as dCt normalized with *RPL32*. (**F**) Comparative quantification analysis of *LRP1* mRNA expression levels in tumor cells compared with stromal cells after LCM. Values are shown as ddCt fold induction. ^*^p < 0.05; ^**^p < 0.01; ^****^p < 0.0001, Mann Whitney test. (**G**) Linear regression analysis of *LRP1* mRNA expression levels evaluated by qRT-PCR on complete fresh frozen adenocarcinoma sample against LRP1 IHC score of tumor cells obtained by multiplying staining intensity (0 to 3) and percentage of positive cells (0 to 4). (**H**) Linear regression analysis of *LRP1* mRNA expression of tumor cells against LRP1 IHC score of tumor cells obtained by multiplying staining intensity (0 to 3) and percentage of positive cells (0 to 4) after LCM.

Owing to the difference of LRP1 IHC expression between stromal and malignant cells, we performed Laser Capture Microdissection (LCM) analyses to distinguish between *LRP1* mRNA expression arising from malignant and stromal cells. LCM was performed on available fresh frozen samples of 32 colon adenocarcinomas. The efficiency of LCM for separating malignant and stromal cell was ensured morphologically (Figure 2A and 2B) and by mRNA quantification of the epithelial marker *carcinoembryonic antigen* (*CEA*) (Figure 2C and 2D). LCM analyses revealed that *LRP1* mRNA expression was 5.1-fold lower in adenocarcinoma cells than in stromal cells (Figure 2E and 2F).

Tumor IHC scores were not correlated with *LRP1* mRNA expression levels of whole adenocarcinoma samples (*p* = 0.10; *R*^2^ = 0.02) (Figure 2G) but were correlated with *LRP1* mRNA expression in tumor cells obtained after LCM (*p* = 0.003; *R*^2^ = 0.28) (Figure 2H). Thus, overall *LRP1* mRNA expression does not reflect malignant cells expression but reflects the sum of malignant and non-malignant cells expression.

### Adenocarcinomas with low LRP1 immunohistochemical expression have a distinct clinicopathological and molecular phenotype

**Figure 3:**
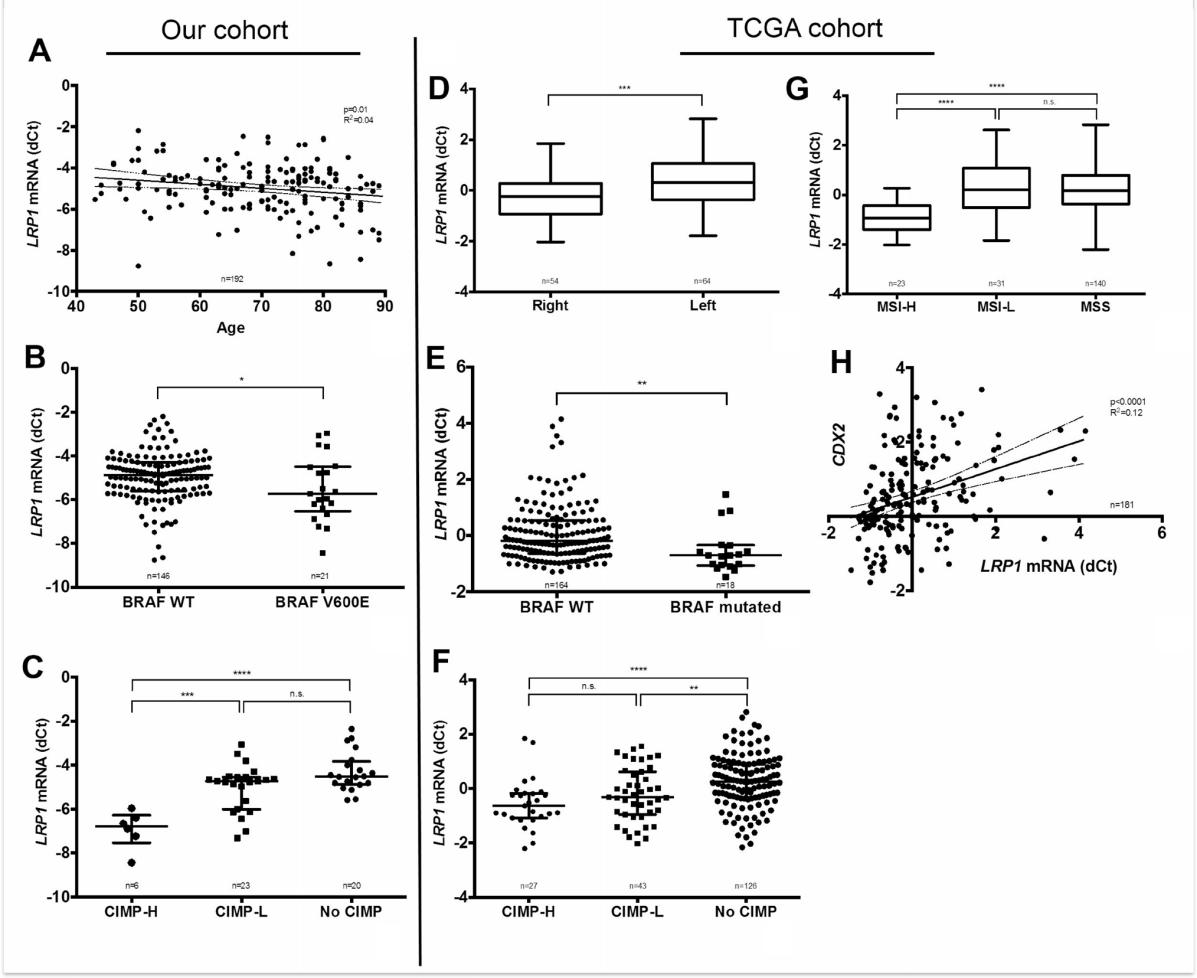
Correlation of *LRP1* mRNA levels with clinical and molecular findings Left panel: *LRP1* mRNA levels analyses by qRT-PCR (dCt normalized with *RPL32*) on fresh frozen colon adenocarcinoma samples from our cohort compared with age (**A**), *BRAF*V600E mutation (**B**) and CpG island methylator phenotype (CIMP-H) (**C**). Right panel: Correlation analysis of *LRP1* mRNA expression levels extracted from the colorectal cancer cohort of the TCGA, as retrieved using cBioportal for Cancer Genomics (http://cbioportal.org) web resources with sided adenocarcinomas (**D**), *BRAF* mutation (**E**), CIMP status (**F**), MSI status (**G**). and *CDX2* mRNA expression (**H**). ^*^p < 0.05; ^**^p < 0.01; ^***^p < 0.001; ^****^p < 0.0001, Mann Whitney test. Abbreviations: H, high; L, Low; MSI, microsatellite instability; MSS, microsatellite stability; CIMP, CpG island methylator phenotype.

Relationship between clinico-pathological and molecular parameters with LRP1 immunohistochemical score in malignant and stromal cells were evaluated in our cohort. As detailed in Table 2, colon adenocarcinomas with low tumor IHC score were associated on univariate analyses with female gender, higher age, right location, high differentiation grade, mucinous type, Annexin A10 expression, loss of CDX2 expression, MSI-H status, *BRAF*V600E mutation, absence of *KRAS* mutation and CIMP-H. On multivariate analyses, low LRP1 IHC score in tumor cells was associated with right location (*p* = 0.0004), MSI-H (*p* = 0.01) and *BRAF*V600E mutation (*p* = 0.009). Moreover, IHC results on tumor cells were confirmed at the mRNA level by qRT-PCR for age (*p* = 0.01), *BRAF*V600E mutation (*p* = 0.05) and CIMP-H phenotype (*p* < 0.001) (Figure 3A–3C).

**Table 2:** Clinicopathological characteristics associated with low LRP1 immunohistochemical expression in adenocarcinomatous cells

	*n*	Mean LRP1 tumor score	*p* univariate	*p* multivariate
**Sex**	**307**		**0.0006 ‡**	n.s
Male	174	5.64 ± 4.01		
Female	133	4.03 ± 4.03		
**Age**	**307**		**0.0009** ‡	0.09
≤ 71 years	140	5.78 ± 4.2		
> 71 years	167	4.24 ± 3.89		
**Tumor location**	**307**		**<0.0001 ‡**	**0.0004**
Right	136	3.34 ± 3.60		
Left	171	6.22 ± 4.02		
**UICC stage**	**304**		0.20 †	NA
Stage I	35	6.2 ± 3.53		
Stage II	115	5.07 ± 4.37		
Stage III	79	4.76 ± 4.12		
Stage IV	75	4.45 ± 3.79		
**Vascular invasion**	**300**		0.30 ‡	NA
Yes	129	4.71 ± 4.1		
No	171	5.2 ± 4.07		
**Perineural invasion**	**300**		0.51 ‡	NA
Yes	87	4.75 ± 3.97		
No	213	5.09 ± 4.14		
**Budding score**	**286**		0.49 **‡**	NA
High	14	4.21 ± 4.28		
Low	272	4.99 ± 4.08		
**Differentiation grade**	**307**		**<0.0001 ‡**	n.s
Grade 1-2	258	5.44 ± 4.03		
Grade 3	49	2.35 ± 3.39		
**CDX2**	**303**		**0.0003 ‡**	n.s
Positive	278	5.19 ± 3.98		
Negative	25	2.16 ± 4.19		
**Mucinous type**	**287**		**0.004 ‡**	n.s
Yes	19	2.37 ± 3.58		
No	268	5.14 ± 4.06		
**Annexin A10**	**305**		**<0.0001 ‡**	n.s
Positive	39	1.90 ± 3.31		
Negative	266	5.41 ± 4.01		
***KRAS* status**	**149**		**0.003 ‡**	n.s
Wild type	101	3.35 ± 3.72		
Mutant	48	5.31 ± 3.82		
***BRAF* status**	**303**		**<0.0001 ‡**	**0.009**
Wild type	259	5.5 ± 3.93		
Mutant	44	1.29 ± 2.69		
**Microsatellite status**	**305**		**<0.0001 ‡**	**0.01**
MSS	265	5.52 ± 3.96		
MSI	40	1.1 ± 2.47		
**CIMP status**	**62**		**0.02 †**	NA
No CIMP	23	5.09 ± 3.94		
CIMP-Low	32	3.75 ± 4.54		
CIMP-High	7	0 ± 0		

NA : Not adopted ; n.s : not significant; ‡ *T* test † Linear regression.

Furthermore, low LRP1 stromal IHC score was associated on univariate analyses with younger age, UICC stage and mucinous type as detailed in Table 3. On multivariate analysis, low LRP1 stromal IHC score was associated with younger age only.

**Table 3:** Clinicopathological characteristics associated with LRP1 immunohistochemical expression in stromal cells

	*n*	Mean LRP1 stromal score	*p* univariate	*p* multivariate
**Sex**	**307**		0.20‡	NA
Male	174	10.80 ± 2.37		
Female	133	11.14 ± 2.22		
**Age**	**307**		**0.03**‡	**0.004**
≤71 years	140	10.63 ± 2.63		
>71 years	167	11.22 ± 1.96		
**Tumor location**	**307**		0.53 ‡	NA
Right	136	11.04 ± 2.20		
Left	171	10.88 ± 2.39		
**UICC stage**	**304**		**0.02 †**	n.s
Stage I	35	9.94 ± 3.07		
Stage II	115	11.16 ± 2.11		
Stage III	79	11.30 ± 1.75		
Stage IV	75	10.85 ± 2.44		
**Vascular invasion**	**300**		0.24 ‡	NA
Yes	129	11.12 ± 2.08		
No	171	10.81 ± 2.47		
**Perineural invasion**	**300**		0.61‡	NA
Yes	87	10.84 ± 2.31		
No	213	10.99 ± 2.32		
**Budding score**	**286**		0.41 **‡**	NA
High	14	10.21 ± 3.24		
Low	272	10.96 ± 2.27		
**Differentiation grade**	**307**		0.17 ‡	NA
Grade 1–2	258	10.83 ± 2.47		
Grade 3	49	11.51 ± 1.41		
**CDX2**	**303**		0.32 ‡	NA
Positive	278	10.36 ± 3.07		
Negative	25	10.99 ± 2.24		
**Mucinous type**	**287**		**0.003 ‡**	n.s
Yes	19	11.68 ± 0.94		
No	268	10.87 ± 2.38		
**Annexin A10**	**305**		0.81 ‡	NA
Positive	39	11.02 ± 2.03		
Negative	266	10.93 ± 2.36		
***KRAS* status**	**149**		0.11 ‡	NA
Wild type	101	11.22 ± 2.06		
Mutant	48	10.60 ± 2.39		
***BRAF* status**	**303**		0.89 ‡	NA
Wild type	259	10.97 ± 2.32		
Mutant	44	11.02 ± 2.10		
**Microsatellite status**	**305**		0.81 ‡	NA
MSS	265	10.93 ± 2.34		
MSI	40	11.02 ± 2.18		
**CIMP status**	**62**		0.36 †	NA
No CIMP	23	10.61 ± 3.00		
CIMP-Low	32	11.28 ± 1.59		
CIMP-High	7	10.00 ± 3.46		

NA : not adopted ; n.s : not significant; ‡ *T* test † Linear regression.

Thus, LRP1 IHC score on tumor cells was associated with peculiar clinicopathological and molecular characteristics. Despite an inverse correlation between tumor and stromal cells IHC scores, these peculiar characteristics were not found in stromal cells.

To further confirm our results in an independent patients’ cohort, associations between *LRP1* mRNA expression levels and available clinical and molecular characteristics were studied in the TCGA cohort (*n* = 212) [36]. As in our cohort, *LRP1* mRNA expression levels among the TCGA cohort were significantly lower in cases with right tumor location (*p* = 0.0003), MSI-H (*p* < 0.0001), *BRAF* mutation (*p* = 0.0015) CIMP-H (*p* < 0.0001) and low *CDX2* expression (*p* < 0.0001) (Figure 3D–3H). In this cohort, LRP1 mRNA expression levels were not associated with patients’ gender, AnnexinA10 (*ANXA10*) expression and KRAS mutation.

In summary, low LRP1 expression evaluated by immunohistochemistry and by qRT-PCR in two independent cohorts is strongly associated with right tumor location, MSI-H, *BRAF* mutation and CIMP-H. These characteristics are those of the hypermutated type of the TCGA [36]. Right colonic cancers with this molecular subtype of CRC are known to have a poor prognosis [32–35].

### Low LRP1 immunohistochemical expression in tumor cells correlates with poor overall survival

**Figure 4:**
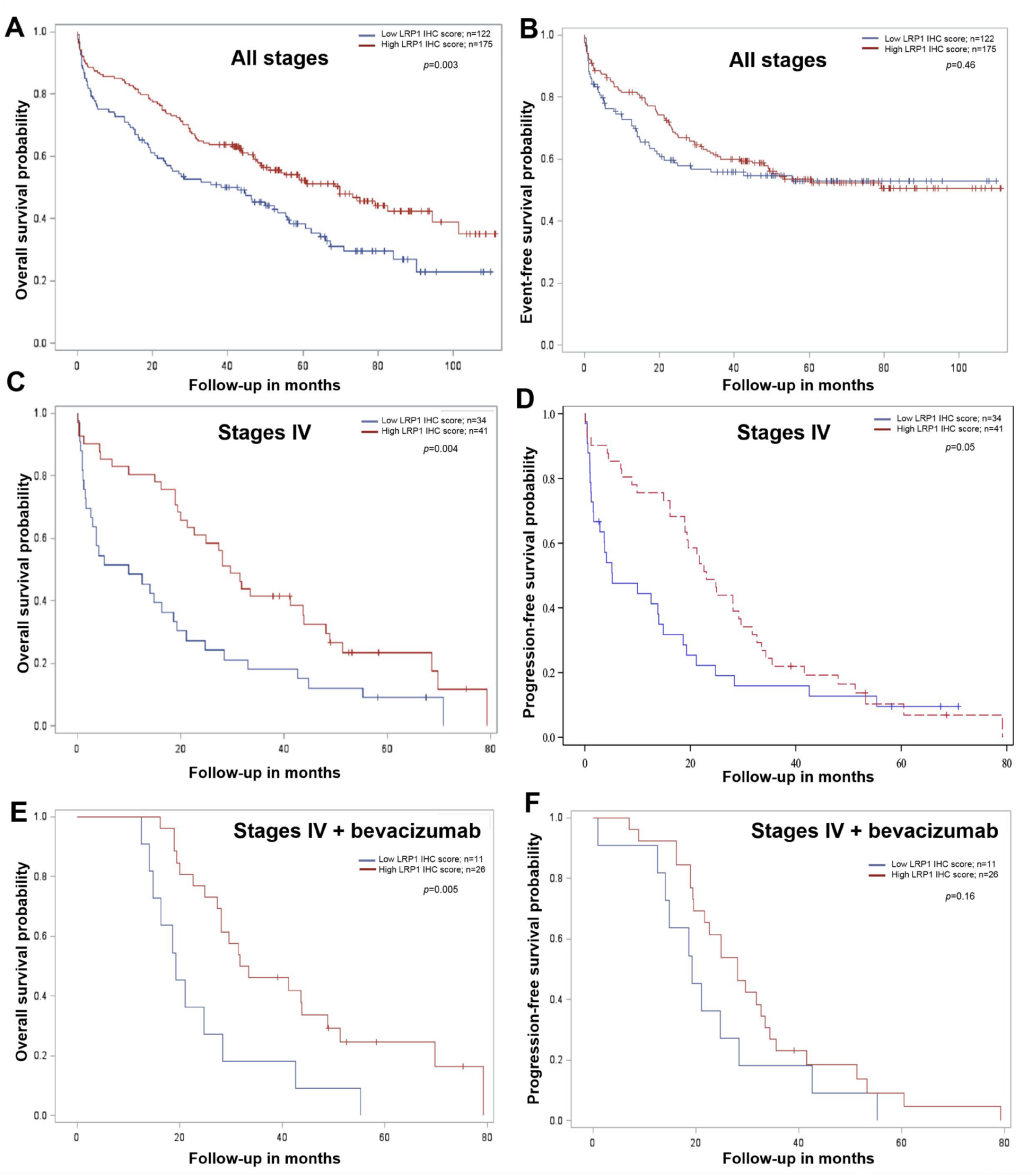
Survival analysis in colon cancer patients from our cohort compared with LRP1 immunohistochemical expression in tumor cells Kaplan-Meier curves of overall survival and event or progression free-survival probability for low (red line) and high (blue line) LRP1 immunohistochemical (IHC) score in adenocarcinoma cells whatever the tumor stage (**A**, **B**), in stage IV (metastatic) patients (**C**, **D**) and in stage IV patients treated with bevacizumab (**E**, **F**). IHC score were evaluated by multiplying staining intensity (0 to 3) and percentage of positive malignant cells (0 to 4) obtained with anti-LRP1 clone 8G1 immunolabelling. Median IHC score was used to separate low (score 0 to 4) and high (score 6 to 12) LRP1 IHC score. All *p* values were calculated using the log rank test.

We subsequently analyzed the relation between LRP1 expression and prognosis. As detailed in Table 4, univariate analysis in our cohort revealed that age, metastatic status, histological grade, vascular invasion, perineural invasion and CDX2 expression were predictors of overall survival (OS). Low LRP1 IHC score in tumor cells (score ≤ 4) was predictor of poor OS (*p* = 0.003) (Figure 4A and Table 4). The value of LRP1 IHC score in tumor cells nearly reached statistical significance as a prognosis indicator of OS (*p* = 0.09) in multivariate analyses.

**Table 4:** Univariate and multivariate analyses of factors associated with overall and event-free survival in our entire cohort of 307 patients

Variables	Overall Survival				Event Free Survival			
	Univariate		Multivariate		Univariate		Multivariate	
	*p* value	HR	95%CI	*p* value	*p* value	HR	95%CI	*p* value
Age	**0.005**	1.03	1.01–1.05	**0.0004**	0.37			N.A
Metastasis (M0 *vs.* M+)	**<0.0001**	2.10	1.40–3.13	**<0.0001**	**<0.0001**	1.57	1.01–2.45	**0.04**
Vascular invasion (yes *vs*. no)	**<0.0001**	1.56	1.10–2.23	**0.01**	**<0.0001**	1.89	1.17–3.02	**0.008**
Perineural invasion (yes *vs*. no)	**0.002**			n.s	**<0.0001**			n.s
Differentiation grade (3 *vs*. 1-2)	**0.003**			n.s	**0.003**			n.s
CDX2 IHC expression (yes *vs*. no)	**0.0005**	1.59	0.93–2.72	0.09	0.11			N.A
*KRAS* mutation (yes *vs.* no)	0.22			N.A	**0.003**	1.62	1.06–2.49	**0.03**
LRP1 IHC tumor score (low *vs.* high)	**0.003**	1.35	0.95–1.93	0.09	0.46			N.A
LRP1 IHC stroma score (low *vs.* high)	0.42			N.A	0.92			N.A
*LRP1* mRNA	0.12			N.A	0.59			N.A

n.s: not significant; N.A: not adopted; HR: hazard ratio. Results were adjusted on T and N.

Metastatic status, vascular invasion and *KRAS* mutation were the only independent predictors of event free survival (EFS) in our cohort (Table 4, Figure 4B).

In our cohort, *LRP1* mRNA expression levels were not correlated with OS and EFS (Table 4).

Stage-specific analyses revealed that LRP1 expression was not a survival predictor of both OS and EFS in UICC stage II and III patients (data not shown). Furthermore, as detailed in Table 5, in metastatic patients (stage IV, *n* = 76), low LRP1 IHC score in malignant cells was an independent predictor of poor OS on univariate (*p* = 0.004, Figure 4C) and multivariate (*p* = 0.03) analyses. Low LRP1 IHC score in malignant cells was predictor of shorter progression-free survival (PFS) on univariate analyses only (Table 5, Figure 4D).

**Table 5:** Univariate and multivariate analyses of factors associated with overall and progression survival in 76 UICC stage IV patients

Variables	Overall survival				Progression free survival			
	Univariate		Multivariate		Univariate		Multivariate	
	*p* value	HR	95%CI	*p* value	*p* value	HR	95%CI	*p* value
Age	**0.05**	1.03	1.00–1.05	**0.04**	0.52			N.A
Tumor location (right *vs.* left)	**0.01**			n.s	**0.01**			n.s
Occlusion (yes *vs*. no)	**0.003**	1.96	1.40–3.69	**0.04**	**0.006**			n.s
Tumor perforation (yes *vs*. no)	0.10	2.23	1.02–4.86	**0.04**	0.07			n.s
Differentiation grade (3 *vs*. 1-2)	**0.0002**	2.18	1.07–4.17	**0.03**	**<0.0001**	3.45	1.73–6.88	**0.0004**
LRP1 IHC tumor score (high *vs.* low)	**0.004**	0.55	0.32–0.96	**0.03**	**0.05**			n.s
LRP1 IHC stroma score (high *vs.* low)	0.11			N.A	**0.03**	2.58	1.35–4.94	**0.004**
*LRP1* mRNA	0.12			N.A	0.22			N.A

n.s: not significant; N.A: not adopted; HR: hazard ratio. Results were adjusted on T and N.

Among stage IV patients from our cohort with available information regarding medical treatment, 48 received 5-fluorouracil-based chemotherapies (LV5FU2, 9; FOLFOX, 14; FOLFIRI, 24; FOLFIXIRI, 1). The most frequently used targeted therapy was the Vascular Endothelial Growth Factor (VEGF) inhibitor bevacizumab (37/76), followed by the Epidermal Growth Factor Receptor (EGFR) inhibitors cetuximab (10/76) and panitumumab (7/76). In patients treated with bevacizumab (*n* = 37), low LRP1 IHC score in tumor cells was associated with shorter OS (Figure 4E). However, low LRP1 IHC tumor score was not predictor for PFS in these patients (Figure 4F).

High LRP1 IHC score in stromal cells was predictor of shorter PFS only on both univariate and multivariate analyses in stage IV patients only (Tables 4 and 5).

**Figure 5:**
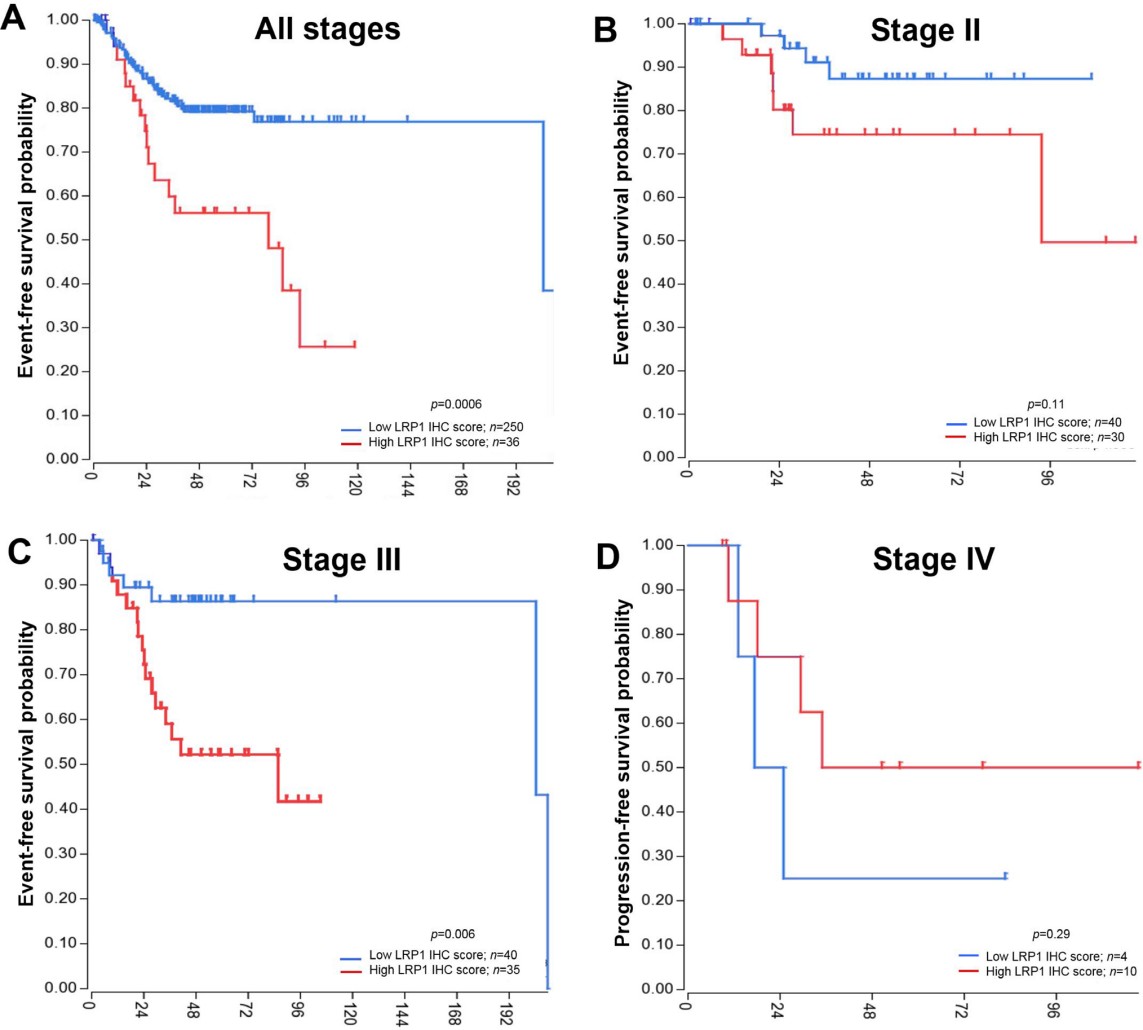
Event-free survival analyses in an independant cohort Publicly available SieberSmith gene expression dataset was obtained from R2 microarray analysis and visualization platform (http://r2.amc.nl), and used for survival analyses. Event-free survival Kaplan-Meier curves for *LRP1* mRNA expression in all stages **(A**), in stage II (**B**) and in stage III patients (**C**). **(D**) Progression-free survival Kaplan-Meier curve for *LRP1* mRNA expression in stage IV patients. All *p* values were calculated using the log rank test and computed using R2 online tools.

To confirm our results, we performed survival analyses in the SieberSmith cohort (*n* = 286) from R2 database [37, 38]. In this cohort, high *LRP1* mRNA expression was a poor prognostic predicator for EFS (*p* = 0.0006) in the entire cohort (Figure 5A). Stage-specific analyses in this cohort revealed that LRP1 was an indicator of EFS in stage III patients only (Figure 5B–5D). In this patient’s group (*n* = 75), high LRP1 expression was associated with shorter EFS (*p* = 0.006). OS data were not available for this cohort.

Despite apparent conflicting results of LRP1 IHC and mRNA survival analyses, LRP1 expression was found to be a strong prognosis indicator. IHC analyses allowed to distinguish LRP1 expression between malignant and stromal cells. In our cohort, LRP1 IHC score in malignant cells was a strong prognosis indicator for OS especially in stage IV patients, whereas LRP1 IHC score in stromal cells was an indicator of PFS in stage IV patients only.

In the SieberSmith cohort, overall *LRP1* mRNA expression may have little significance because it reflects the ratio of epithelial cells (normal and malignant) *versus* non-epithelial cells.

### Analyses of LRP1 expression regulation by mutation, methylation and microRNA

**Figure 6:**
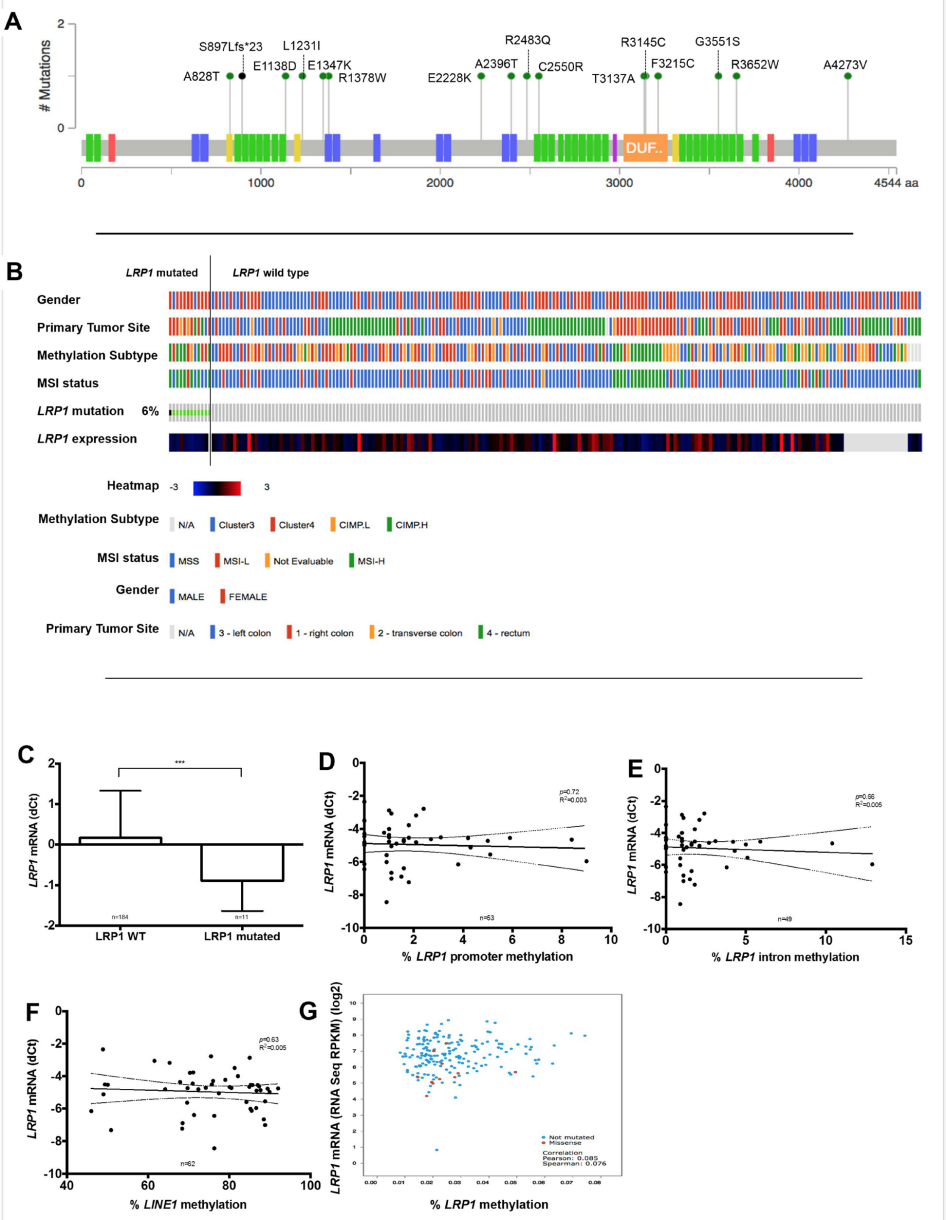
Analysis of LRP1 expression regulation by *LRP1* gene mutation or methylation (**A**) Somatic mutation data from the complete length of *LRP1* gene obtained from colorectal cancer of the TCGA cohort using cBioportal for Cancer Genomics (http://cbioportal.org) web resources. Colored boxes present on the *LRP1* gene representation correspond to exons encoding functional domains of LRP1. Green domain, low-density lipoprotein receptor domains; blue, low-density lipoprotein receptor repeats; yellow, coagulation factor Xa inhibitory site; orange, domain of unknown function; red, calcium-binding EGF domain; violet, complement Clr-like EGF-like. (**B**) Graphical representation of association of *LRP1* mutational status with clinical and molecular tracks and *LRP1* mRNA expression. (**C**) *LRP1* mRNA expression levels comparison between *LRP1* mutated and *LRP1* wild type colorectal cancer. ^***^p = 0.003, Mann Whitney test. Linear regression analyses between *LRP1* mRNA expression levels and promoter methylation (**D**), intronic methylation (**E**), global DNA methylation levels approximated by *LINE1* (**F**) in our cohort. (**G**) Correlation of *LRP1* mRNA expression levels and *LRP1* promoter methylation in data extracted from the TCGA.

In order to explain the decrease of LRP1 expression in malignant cells, we analyzed genetic and epigenetic modifications that could be involved in *LRP1* expression regulation. First, mutation analysis among the TCGA cohort dataset [36] revealed that *LRP1* gene mutation was rare (6%; 12/212), without particular hotspot mutation site (Figure 6A). Then, *LRP1* mutation was strongly associated with female gender (*p* < 0.0001), right tumor location (*p* = 0.04), MSI-H (*p* < 0.0001) and CIMP-H status (*p* = 0.0006) (Figure 6B). Besides, *LRP1* mRNA expression was lower expressed in the *LRP1-*mutated group when compared with *LRP1* wild type group (*p* = 0.003) (Figure 6C). Hence, although infrequent, *LRP1* mutations may partly explain the decrease in *LRP1* mRNA expression in some CRC.

Due to the low rate of *LRP1* mutation, it is likely that other phenomenon, such as epigenetic modifications, may be involved in *LRP1* gene expression regulation. To explore *LRP1* epigenetic modifications, we analyzed both intronic and promoter methylation on available fresh frozen samples of 64 adenocarcinomas and 39 normal colon mucosa. Surprisingly, *LRP1* promoter or intronic methylation levels were very low in all these samples. Moreover, *LRP1* mRNA expression levels and LRP1 IHC score in tumor cells were neither correlated with *LRP1* intronic or promoter levels nor with global methylation as evaluated by *LINE1* methylation levels (Figure 6D–6F).

Available *LRP1* methylation analyses from the TCGA cohort (*n* = 212) [36] confirm the low level of *LRP1* gene methylation (Figure 6G). In this cohort, no correlation was found between *LRP1* mRNA expression and *LRP1* methylation levels (*p* = 0.08).

Thus, *LRP1* methylation does not seem to be involved in the regulation of *LRP1* gene expression.

**Figure 7:**
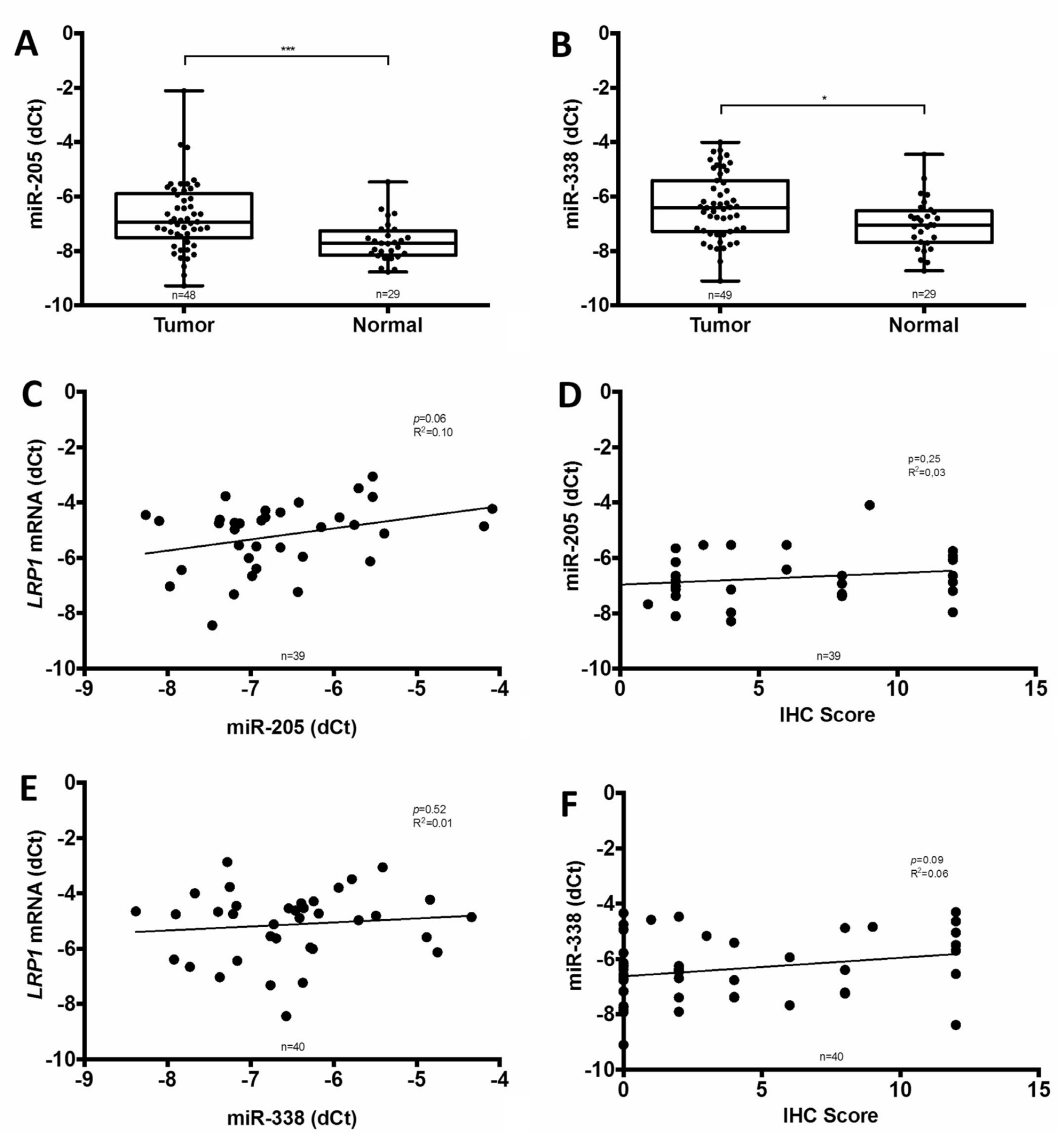
Comparison of miR-205 and miR-338-5p expression with LRP1 expression Analyses of miR-205 (**A**) and miR-338-5p (**B**) expression by qRT-PCR in fresh frozen colon cancer adenocarcinoma compared with normal colon mucosa from our cohort (^*^p < 0.05, ^***^p < 0.001, Mann Whitney test). Linear regression analysis of *LRP1* mRNA expression levels evaluated by qRT-PCR on complete fresh frozen adenocarcinoma sample against miR-205 (**C**) and miR-338-5p (**E**) expression. Linear regression analysis of miR-205 (**D**) and miR-338-5p (**F**) expression against LRP1 immunohistochemical (IHC) score of tumor cells. IHC score was assessed by multiplying staining intensity (0 to 3) and percentage of positive tumor cells (0 to 4) with anti-LRP1 clone 8G1 immunolabelling.

To evaluate the putative contribution of miRNA, two of the most important miRNA implicated in *LRP1* expression regulation i.e. miR-205 and miR-338-5p were assessed on available fresh frozen samples of 49 adenocarcinomas and 29 paired normal colon mucosa. In these samples, both miR-205 and miR-338-5p were significantly higher expressed in adenocarcinomas than in normal colon (Figure 7A and 7B). Moreover, linear regression analyses revealed that miR-205 tended to stimulate *LRP1* mRNA expression (*p* = 0.06; *R*^2^ = 0.10) (Figure 7C) despite the absence of correlation with LRP1 IHC score in tumor cells (Figure 7D). Additionally, no correlation was found between *LRP1* mRNA level or IHC score in tumor cells and miR-338-5p expression (Figure 7E and 7F). Thus, miR-205 expression does not appear to be implied in the low expression of LRP1 in adenocarcinomatous cells.

## DISCUSSION

LRP1 has been attributed a role in cancer. Such multifunctional endocytic receptor has both endocytic and signaling activities. LRP1 expression levels are often dysregulated in cancer, while LRP1 role varies from one tumor type to another. In CRC, the role and impact of LRP1 expression remained however unknown so far.

In this report, we made several important and previously unrecognized findings regarding the role of LRP1 in colon cancer. First, qRT-PCR, LCM and IHC analyses revealed that LRP1 was significantly lower expressed in adenocarcinoma cells than in normal mucosa and stromal cells. Second, analysis of two independent patient cohorts revealed that low LRP1 expression correlated with right tumor location and specific molecular profile. Third, low LRP1 IHC score in tumor cells was associated with poor OS in non-metastatic colon cancer patients, and was an important prognostic and predictive factor in metastatic patients. Finally, we found that LRP1 expression could be partly regulated by *LRP1* mutation.

In our cohort, LRP1 was lower expressed, both at mRNA and protein levels, in malignant cells compared with colonic mucosa and stromal cells. In colon mucosa, we observed that LRP1 expression seems to be restricted to surface epithelium, which is the most specialised part of the epithelium. However, the surface epithelium is about to undergo apoptosis. Thus, staining of these cells should be interpreted with caution. Some cells of the lamina propria, especially myofibroblasts, expressed LRP1 as previously described [30, 31]. In adenocarcinomas, IHC and LCM analyses highlighted the differential expression pattern of LRP1 between tumor and stromal cells. Such a loss of LRP1 expression in tumor cells as well as its strong expression in stromal fibroblast were previously described in small cohorts of CRC [30, 31] and in other types of cancer such as pancreatic ductal adenocarcinoma [39] and lung adenocarcinoma [21]. These previous studies showed that this differential expression between tumor and stromal cells seems to play a role in tumor aggressiveness. In pancreatic carcinoma, high stromal expression of LRP1 was correlated with a decreased activation of caspase 3 in tumor cells and increased level of SNAIL, a transcription factor promoting epithelial-mesenchymal transition and cell migration [39]. In CRC, high stromal expression of LRP1 was correlated with high u-PA expression in stromal cells [30]. In hepatocellular carcinoma and Wills tumor cells, the diminished expression of LRP1 in tumor cells correlated with increased levels of MMP9, probably due to loss of LRP1-mediated endocytosis [20, 23]. Thus, the differential expression of LRP1 between tumor and stromal cells might confer survival and spreading benefits for tumor cell in some tumor types including CRC.

The loss of LRP1 expression in tumor cells is partly explained by mutations in *LRP1* gene. Indeed, we observed a loss of LRP1 IHC expression in 21% of the cases in our cohort, while *LRP1* gene mutation only occurred in 6% of the TCGA cohort cases. These *LRP1*-mutated cases shared the same clinical and molecular profile as those with low LRP1 IHC score in tumor cells and low *LRP1* mRNA expression: right location, MSI-H and CIMP-H. Right colonic cancers with this molecular pattern correspond to the hypermutated type of the TCGA molecular type of CRC [36]. Hypermutated CRC had a higher mutation rate than non-hypermutated CRC [36], this being mainly due to mismatch repair system deficiency related to *MLH1* methylation. Thus, in this molecular subgroup of CRC, loss of LRP1 expression can partly be explained by *LRP1* gene mutation. *BRAF* mutation is found in around 80–90 % of sporadic MSI-H colorectal cancers [36]. Thus, low *LRP1* mRNA might be correlated to *BRAF* mutation through the hypermutator type of CRC. Furthermore, hypermutated CRC are also known as displaying frequent gene hypermethylation. Due to the abundance of CpG islands in *LRP1* gene promoter and frequent hypermethylation of *LRP1B*, another member of the LRP family, in various cancer types [40, 41], it could be possible that *LRP1* gene methylation might regulate its expression. However, in our cohort as well as in the TCGA cohort, the methylation level of both intronic and promoter region of *LRP1* was very low, suggesting that epigenetic regulation by methylation was not involved in the regulation of *LRP1* expression.

We therefore investigated the role of microRNAs (miRNAs) regulation on LRP1 expression. Previous studies on vascular smooth muscle cells, glioma and lung carcinoma cells showed that expression of LRP1 was negatively regulated by miR-205 [42, 43]. This reduced expression of LRP1 by miR-205 led to decreased tumor cell migration [42]. In colon cancer, miR-205 expression findings are conflicting. In one study [44], miR-205 was higher expressed in colon cancer than in paired normal colon. Another study found inverse results [45]. These studies also found conflicting results regarding the role of miR-205 in regulation of cell proliferation [44, 45]. Moreover, contrary to previous studies, we found that miR-205 tended to regulate LRP1 expression positively. However, this correlation is weak. Thus, miR-205 could regulate LRP1 expression in colon cancer but its precise role needs to be furher clarified.

LRP1 IHC score and *LRP1* mRNA expression after LCM in malignant cells were correlated. Thus, pre-transcriptional processes seem to be involved in LRP1 down-regulation. Indeed, in our study, *LRP1* mutation that is found in 6% of the cases might partly explain the loss of LRP1 expression observed in 21% of the cases. *LRP1* methylation and miR-205 do not seem to be involved in LRP1 expression down-regulation. Thus, other epigentic pre-transcriptional processes might be involved such as regulation by other miRNA, by transcriptional factors or by histone methylation or acethylation. Some microRNA might directly target effectors of the epigenetic machinery (such as DNA methyltransferases, histone deacetylases, and polycomb repressive complex genes) and indirectly affect the expression of tumor suppressor genes [46].

In colon cancer, the low expression of LRP1 in tumor cells was strongly associated with right tumor location, poor differentiation, *BRAF* mutation, MSI-H and CIMP-H status in our cohort as well as in an independent CRC cohort. These molecular findings correspond to the hypermutated subtype of the TCGA classification [36] and the MSI-immune subtype according to the Consensus Molecular Subtype consortium [32]. These subtypes were found in several studies to be associated with serrated pathway and to have a poor prognosis, particularly after relapse [32–35]. In our cohort, low LRP1 IHC score in tumor cells was associated with poor OS particularly in metastatic (stage IV) patients. Inverse results were found in the SieberSmith cohort, in which *LRP1* expression was assessed by qRT-PCR. In this cohort, low *LRP1* mRNA expression was related to better EFS. However, mRNA expression reflects combined stromal and tumor cells expression. Conversely, our LCM analyses showed first that LRP1 was overexpressed in stromal cells when compared with tumor cells and second that *LRP1* mRNA expression in tumor cells obtained by LCM were correlated to LRP1 IHC score on tumor cells. Thus, *LRP1* mRNA expression levels on whole tumor samples is more likely to reflect stromal cell expression rather than being representative of tumor cell expression. Moreover, in our cohort, high stromal LRP1 IHC expression in stage IV patients was associated with poor PFS. Thus, the results of the SieberSmith cohort might more reflect the prognosis impact of LRP1 expression in stromal cells. So, we think that *LRP1* mRNA expression results obtained from the SieberSmith cohort should not be completely superimposed with our IHC findings. In addition, the IHC score on tumor cells can be easily and routinely performed on formalin-fixed and paraffin-embedded CRC tissue, while mRNA analyses requires high quality fresh frozen tissue. Thus, from a practical point of view, LRP1 IHC score assessed in malignant cells seems to be more informative for clinical outcome rather than global mRNA expression. Other studies are needed to clarify our results regarding LRP1 IHC expression in malignant and stromal cells.

To date, the biologic agents that have been proven as having clinical benefits in metastatic CRC mainly target VEGF and EGFR. In particular, bevacizumab targeting VEGF and cetuximab or panitumumab targeting EGFR have demonstrated significant survival benefits in combination with cytotoxic chemotherapy in first-line, second-line, or salvage setting. However, recent retrospective analyses have shown that *KRAS* or *NRAS* mutations were negative predictive markers for anti-EGFR therapy [47]. The mechanisms of action of anti-VEGF are not completely understood, and apart from right tumor location, no predictive factor has yet been validated [48, 49]. The role of *KRAS* or *NRAS* mutation for bevacizumab therapy efficiency prediction has not been defined yet [50]. In our study, low LRP1 IHC score in tumor cells was an indicator of poor OS and PFS in metastatic patients. Indeed, stage IV patients with low LRP1 IHC score in tumor cells had shorter OS, even when treated with bevacizumab. However, our results in metastatic patients are limited by the small number of patients treated with bevacizumab in our cohort (*n* = 37) and the lack of *NRAS* status data. Nevertheless, in view of our promising results, we believe the potential role of LRP1 IHC for predicting bevacizumab benefit in metastatic CRC patient needs to be studied in larger and prospective cohorts.

The prognosis impact of low LRP1 IHC expression in malignant cells from stage IV patients may only be partly explained by its association with microsatellite instability. Indeed, stage II-III MSI CRC had a better prognosis than stage II-III MSS CRC. However, stage IV MSI CRC are associated with poor prognosis and chemoresistance, especially to 5FU-based chemotherapy [51]. Thus, the association of low LRP1 expression with MSI might explain the pejorative prognosis impact of low LRP1 expression on metastatic patients only. Moreover, a recent study had shown that stage IV CRC that were non-responders to bevacizumab therapy had a higher level of MMP12 expression than responders [52]. This increase in MMP12 expression may be favored by the decrease of LRP1 expression. However, this hypothesis remains to be demonstrated.

In summary, our study show that low LRP1 IHC expression in malignant colon adenocarcinoma cells is a strong prognosis predicator, especially in metastatic patients, in which it predicts a shorter OS in patients treated by anti-VEGF therapies. The lower expression of LRP1 in malignant cells is partly explained by *LRP1* gene mutation through the hypermutator type of CRC.

## MATERIALS AND METHODS

### Patients

The study was conducted on adult patients who underwent surgery for sporadic colon cancer in the Digestive Surgery Department of the Academic Hospital of Reims between September 2006 and December 2012. Patients with rectal cancer were excluded. All patients had given their consent for biospecimen use. The study was performed in accordance with the ethical standards laid down in the Declaration of Helsinki. Written patients’ consent for biospecimen use was obtained in all cases. Approval for the study was previously obtained from the local Institutional Review Board and the Tissue Bank Management Board. Study design was published on clinicaltrials.gov web site in May 2016 (#NCT02788669).

Clinical data including age at the time of surgery, sex, performance status, surgical circumstances (tumor perforation, occlusion), tumor location, synchronous or metachronous metastases, tumor recurrence, treatment, death and pathological and molecular data including adenocarcinoma type, grade and pTNM stage were collected. Patients were classified as having a right colonic cancer if the primary tumor was located in the caecum, ascending colon, hepatic flexure or transverse colon, and left colonic cancer if the tumor site was within the splenic flexure, descending colon, sigmoid colon or rectosigmoid junction. Mismatched repair (MMR) status of tumors was performed by immunohistochemistry with anti-MLH1, PMS2, MSH2 and MSH6 proteins on tissue microarrays, completed when necessary by microsatellite instability analysis, as already reported [53]. Mutations within exon 2 of *KRAS* and of the codon 600 of *BRAF* were detected as previously described [53]. Follow-up data were obtained from oncologist or attending physicians.

### Pathology

All colon adenocarcinomas were classified and subtyped according to The World Health Organization criteria [1] and staged according to the International Union Against Cancer 2009 guidelines [4]. All slides were retrieved from the archives of the Department of Pathology of the Academic Hospital of Reims and were reviewed and classified by two pathologists (CBR and MDD). Tumor budding was assessed on Hematoxylin-Eosin-Saffron slides as previously described [54].

### Immunohistochemistry

All tissue samples were analyzed *via* tissue microarrays. For each tumor, 3 cores were punched in the central part and 3 cores at the invasive front of the tumor from the same original formalin-fixed paraffin-embedded tumor block. The cores were precisely arrayed into a recipient paraffin block using the MiniCore Tissue Arrayer (Excilone, Elancourt, France). Sections of 4-μm thickness were cut and mounted on SuperFrost Plus Gold adhesive slides (Thermofisher Scientific, Waltham, MA, USA). Immunohistochemistry using anti-LRP-1 α-chain (1/1000, mouse, clone 8G1, Merck, Darmstadt, Germany) and control isotype mouse IgGs (Agilent Technologies, Santa Clara, CA, USA) was performed using Novolink Polymer Detection System (Leica Biosystems, Wetzlar, Germany) after heat-induced epitope retrieval in citrate pH 6 buffer (95° C, 40 min) and overnight antibody incubation at 4° C.

Staining intensity (SI) was graded by two pathologists (CBR, AMB) as 0 (negative), 1 (weak), 2 (moderate) and 3 (strong). The percentage of positive cells (PPC), was graded as follows: 0 (<5%), 1 (5–25%), 2 (26–50%), 3 (51–75%) and 4 (76–100%). In case of discrepancies a consensus diagnosis was reached. Then, an immunostaining score was generated independently for malignant and stromal cells of each case by multiplying SI and PPC. The median score was used to distinguish low (0–4) and high (6–12) LRP1 expression levels for adenocarcinomatous cells.

Additionnaly, imunohistochemistry for the intestinal differentiation marker CDX2 [55] (RTU, rabbit monoclonal, clone EPR2764Y, Zytomed System, Berlin, Germany) and the marker of serrated subtype of adenocarcinoma Annexin A10 [56] (1/400, rabbit polyclonal, Novus Biologicals, Littleton, CO, USA) were performed with the BenchMark XT automated slide stainer (Ventana Medical Systems, Tucson, AZ, USA). Antibody retrieval was performed with Cell Conditioner 1 (EDTA, pH 8.4) incubation for 64 minutes, followed by preprimary peroxidase inhibition, and incubation with the corresponding antibody at 37° C for 32 minutes. UltraView Universal DAB v3 Kit (Ventana Medical Systems) was used for staining reaction. For all immunohistochemistry, the counterstain used was hematoxylin. Staining was rated binarly as either positive or negative for these 2 markers by the same pathologists. All tumors in which the tumor cells completely lacked immunostaining were scored as negative. Cases were rated as positive when the tumor cells were unequivocally stained in the nucleus.

### *LRP1* mRNA analyses

mRNA analyses were performed on fresh frozen colon adenocarcinoma and normal colon tissues sampled on colectomies received at the Pathology Department of Reims University Hospital (France) and stored in the Champagne-Ardenne Biobank as previously described [57]. Total RNAs were isolated and purified with Maxwell^®^ 16 LEV simply RNA tissue kit (Promega, Madison, USA) according to the manufacturer’s instructions on the Promega’s robotics platform Maxwell^®^ 16 Research Instrument (Promega, Madison, USA). The concentration of total RNA (ng/μL) was determined by a Picodrop uL spectrophotometer (Picodrop, Hinxton, United Kingdom).

RNA quality index (RQI) was determined using the Experion™ automated electrophoresis system (Bio-Rad, Marnes-la-Coquette, France) according to the manufacturer protocol. Only RNA with RQI values ≥5 were used for futher analyses.

RNA were reverse-transcribed using VERSO cDNA kit (Thermo Fisher Scientific, Waltham, MA, USA) according to the manufacturer’s instructions using random hexamer primers. Real-time PCR was performed using an Absolute SYBR Green Rox mix (Thermo Fisher Scientific), on a CFX 96 real time PCR detection system (Bio-Rad). *RS18* and *RPL32* were used for *LRP1* expression normalization. The sequences of the pairs of primers used were: *LRP1* (5′–AGA AGT AGC AGG ACC AGA GGG – 3′ and 3′–TCA GTA CCC AGG CAG TTA TGC - 5′), *CEA* (5′–TTT CTC CCT ATG TGG TCG CTC CAG - 3′ and 3′–AGC AGA TTT TTA TTG AAC TTG TGC _- 5′), *RS18* (5′- GCA GAA TCC ACG CCA GTA CAA −3′ and 3′–GCC AGT GGT CTT GGT GTG CT– 5′) and *RPL32* (5′–CAT TGG TTA TGG AAG CAA CAA A- 3′ and 3′–TTC TTG GAG GAA ACA TTG TGA G-5′). All primers were synthesized by Eurogentec (Eurogentec, Liège, Belgium). PCR conditions were set as 15 min at 95° C, followed by 40 cycles each consisting of 15 s at 95° C (denaturation) and 1 min at 60° C (annealing/extension). The specificity of PCR amplification was checked using a heat dissociation curve from 65° C to 95° C following the final cycle. The cycle threshold (Ct) values were recorded with Bio-Rad CFX Manager^™^ 3.0 software (Bio-Rad).

### Laser capture microdissection

Fresh frozen colon adenocarcinoma specimens were cut into 12 μm serial sections and mounted on PALM membrane slides (Zeiss, Oberkochen, Germany). The slides were immediately stained with cresyl violet from the LCM staining kit (Thermo Fischer Scientific) and laser capture microdissection (LCM) was performed immediately thereafter. Adenocarcinomatous and stromal areas were selected during the LCM procedure by a pathologist (CBR). Laser capture microdissection was performed with the PALM MicroBeam instrument (Zeiss). At least 5 mm^2^ of tumor tissue or stromal tissue were collected from each sample. This required from nine to twelve 12 μm sections.

RNA from tumor and stromal microdissected tissues were isolated and purified with the RNeasy micro kit (Qiagen GmbH, Hilden, Germany) according to the manufacturer’s instructions. RNA concentrations were measured using NanoDrop system (Thermo Fisher Scientific). RT-PCR analyses were performed as detailed above.

### miRNA analyses

miRNA were extracted from fresh frozen colon adenocarcinoma and normal colon tissues using miRNeasy mini kit (Qiagen) according to manufacturer’s instructions. RNA concentrations were measured using a NanoDrop spectrophotometer (Thermo Fisher Scientific). cDNA was synthesized using miScript II RT Kit (Qiagen) in accordance with the manufacturer’s instructions.

Expression of miR-205 and miR-338-5p was determined by real time PCR using an Absolute SYBR Green Rox mix (Thermo Fisher Scientific), on a CFX 96 real time PCR detection system (Bio-Rad) and normalized using U6 small nuclear RNA. miR-205 is known to down-regulate *LRP1* expression [42, 43]. miR-338-5p is not implicated in *LRP1* expression regulation and was used as control as previously described [43]. All primers were purchased as 10x miScript Primer Assay (Qiagen). PCR conditions were 15 min at 95° C, followed by 40 cycles each consisting of 15 s at 95° C (denaturation), 30 s at 55° C (annealing) and 30 s at 70° C (extension). The specificity of PCR amplification was checked using a heat dissociation curve from 65° C to 95° C following the final cycle. The cycle threshold (Ct) values were recorded with Bio-Rad CFX Manager^™^ 3.0 software (Bio-Rad).

### Methylation analyses

All methylation analyses were performed on DNA extracted from fresh frozen tissues with the QIAamp DNA microkit (Qiagen) according to manufacturer’s instructions. Bisulfite conversion was performed with the EZ DNA Methylation gold kit (Zymo Research, Irvine, CA, USA) following manufacturer’s instructions.

The CpG island methylator phenotype (No CIMP, CIMP-Low and CIMP-High) was determined by Methylation Sensitive High Resolution Melting for 5 markers (*MLH1*, *CDKN2A*, *MINT1*, *MINT2*, and *MINT31*) on the LightCycler 480 II High Resolution Melting instrument (Roche, Pleasanton, CA, USA). All primers were synthesized by Eurogentec (Eurogentec). No-CIMP status was defined as no methylated locus, CIMP-Low status as one to three methylated loci, and CIMP-High status as four or five methylated loci as previously described [58].

*LINE-1* methylation analyses were performed by pyrosequencing analysis using the Pyromark Q96MA instrument (Qiagen) as previously described [59]. The average *LINE-1* methylation level was calculated as the mean of the proportions of C (%) at the 3 CpG sites analyzed and this indicated the level of methylation of *LINE-1* elements.

*LRP1* methylation analyses were performed by pyrosequencing using the Pyromark Q96MA instrument (Qiagen). Promoter and intronic region of *LRP1* were amplified from bisulfited DNA using commercialy available primers (Hs_LRP1_01_PM for intronic region, Hs_LRP1_02_PM for promoter region, Qiagen) for PCR amplification and pyrosequencing. PCR amplifications were performed using PyroMark PCR kit (Qiagen) according to manufacturer’s instructions. Methylated and unmethylated converted and unmethylated unconverted controls from the EpiTect PCR Control DNA Set (Qiagen) were used for each experiment. Each experiment was performed in duplicate.

### Data mining and bioinformatic analyses

Mutation and expression data from the colorectal carcinoma dataset of the The Cancer Genome Atlas (TCGA; https://tcga-data.nci.nih.gov) [36] were analyzed using cBioportal for Cancer Genomics (http://cbioportal.org) web resources [37, 38].

Publicly available SieberSmith gene expression dataset was obtained from R2 microarray analysis and visualization platform (http://r2.amc.nl), and used for survival analyses. Cut-off value for separating high and low *LRP1* expression groups was determined by the online algorithm.

### Statistical and survival analyses

Data are here described using mean and standard deviation for quantitative variables and number and percentage for qualitative variables. Factors associated with mRNA and immunohistochemical expression of LRP1 were studied using univariate analysis (Chi2 test, Fisher’s exact test, Student’s *t* test, linear regression or Wilcoxon test, as appropriate) and multivariate analysis (linear regression with stepwise selection, with an exit threshold of 0.10 and factors significant at *p* = 0.10 included). Overall and event-free survivals were studied. The survival curves were established by the Kaplan-Meier method. For each analysis, prognostic factors were identified by univariate analysis using log rank tests and by multivariate analysis using a Cox proportional hazard model. Factors significant at the 0.10 level in univariate analysis were included in a stepwise regression multivariate analysis with entry and removal limits set at 0.10. Statistical analyses were performed with SAS version 9.4 (SAS institute Inc, Cary, North California). For all tests, *p* < 0.05 were considered to be statistically significant.

## Abbreviations

CIMP-H: High CpG Island Methylator phenotype
CRC: Colorectal Carcinoma
EGFR: Epidermal Growth Factor Receptor
IHC: Immunohistochemistry
LCM: Laser Capture Microdissection
MSI-H: High Microsatellite instability
MSS: Microsatellite stable
TCGA: The Cancer Genome Atlas
UICC: Union Internationale Contre le Cancer
VEGF: Vascular Endothelial Growth Factor
